# Use of an Outbred Rat Hepacivirus Challenge Model for Design and Evaluation of Efficacy of Different Immunization Strategies for Hepatitis C Virus

**DOI:** 10.1002/hep.30894

**Published:** 2019-10-11

**Authors:** Erwan Atcheson, Wenqin Li, Carly M. Bliss, Senthil Chinnakannan, Kathrin Heim, Hannah Sharpe, Claire Hutchings, Isabelle Dietrich, Dung Nguyen, Amit Kapoor, Michael A. Jarvis, Paul Klenerman, Eleanor Barnes, Peter Simmonds

**Affiliations:** ^1^ Peter Medawar Building for Pathogen Research University of Oxford Oxford UK; ^2^ Centre for Vaccines and Immunity The Research Institute at Nationwide Children’s Hospital Columbus OH; ^3^ University of Plymouth, Plymouth Science Park Plymouth UK

## Abstract

**Background and Aims:**

The lack of immunocompetent small animal models for hepatitis C virus (HCV) has greatly hindered the development of effective vaccines. Using rodent hepacivirus (RHV), a homolog of HCV that shares many characteristics of HCV infection, we report the development and application of an RHV outbred rat model for HCV vaccine development.

**Approach and Results:**

Simian adenovirus (ChAdOx1) encoding a genetic immune enhancer (truncated shark class II invariant chain) fused to the nonstructural (NS) proteins NS3‐NS5B from RHV (ChAd‐NS) was used to vaccinate Sprague‐Dawley rats, resulting in high levels of cluster of differentiation 8–positive (CD8^+^) T‐cell responses. Following RHV challenge (using 10 or 100 times the minimum infectious dose), 42% of vaccinated rats cleared infection within 6‐8 weeks, while all mock vaccinated controls became infected with high‐level viremia postchallenge. A single, 7‐fold higher dose of ChAd‐NS increased efficacy to 67%. Boosting with ChAd‐NS or with a plasmid encoding the same NS3‐NS5B antigens increased efficacy to 100% and 83%, respectively. A ChAdOx1 vector encoding structural antigens (ChAd‐S) was also constructed. ChAd‐S alone showed no efficacy. Strikingly, when combined with ChAd‐NS, ChAD‐S produced 83% efficacy. Protection was associated with a strong CD8^+^ interferon gamma–positive recall response against NS4. Next‐generation sequencing of a putative RHV escape mutant in a vaccinated rat identified mutations in both identified immunodominant CD8^+^ T‐cell epitopes.

**Conclusions:**

A simian adenovirus vector vaccine strategy is effective at inducing complete protective immunity in the rat RHV model. The RHV Sprague‐Dawley rat challenge model enables comparative testing of vaccine platforms and antigens and identification of correlates of protection and thereby provides a small animal experimental framework to guide the development of an effective vaccine for HCV in humans.

**Abbreviations**
ACK: ammonium–chloride–potassium
CD: cluster of differentiation
ELISpot: enzyme‐linked immunosorbent spot
EOT: end of trial
HCV: hepatitis C virus
IFNγ: interferon gamma
i.u.: infectious units
MID: minimum infectious dose
MVA: modified vaccinia virus ankara
NS: nonstructural
PBMC: peripheral blood mononuclear cell
PBS: phosphate‐buffered saline
RHV: rodent hepacivirus
RT: room temperature
SD: Sprague‐Dawley
vp: viral particles

Hepatitis C virus (HCV) infects 1.75 million people per year, with over 399,000 deaths annually.^1^ Despite direct‐acting antivirals to treat HCV, an effective vaccine is still urgently required, not least because of low diagnosis rates in many populations^2^ and difficulty treating at‐risk groups.^3^ HCV vaccine studies have been hampered by the lack of a small animal model.^4^ A rodent homolog of HCV, related but assigned as separate species in the genus *Hepacivirus*, family Flaviviridae, was recently discovered in New York brown (sewer) rats.^5^, ^6^ Infection of the brown rat (*Rattus norvegicus*) by the rodent hepacivirus (RHV) is the basis for our vaccine model.

RHV in rats has a profile very similar to that of HCV, making it an appropriate surrogate model for vaccine study.^6^ Like HCV, it is hepatotropic, generating persistent viremia detectable for over 1 year,^6^, ^7^ and recapitulates many aspects of liver pathology, including microvesicular and macrovesicular steatosis, lymphoid aggregation, and evidence of biliary epithelial damage.^6^ RHV is predicted to encode similar proteins to HCV and possesses similar genetic structures in the untranslated regions flanking the coding region.^6^

The first clinical trial of HCV vaccine efficacy in humans, using adenoviral and modified vaccinia virus ankara (MVA) vaccines to elicit T‐cell responses against HCV nonstructural antigens, has concluded in the United States (clinical trial no. NCT01436357). A press release suggests the vaccine failed to protect against natural HCV infection in this high‐risk group (people who inject drugs).^8^, ^9^, ^10^ It is now vital to understand why this strategy failed. Reaching this stage has been made difficult by the lack of appropriate animal models for preclinical vaccine development, in particular for testing protective efficacy prior to trial.^4^ An ideal small animal model would be an immunocompetent mouse or rat susceptible to HCV infection, manifesting persistence and liver pathology. Despite great efforts in this area, including development of transgenic^11^ and humanized^4^, ^12^, ^13^ mice, no system until now has met all these requirements.

Most information on potential HCV vaccine efficacy has come from chimpanzees, with antibody‐generating^14^, ^15^ and T cell–based^16^, ^17^, ^18^, ^19^ vaccines showing evidence of protective efficacy. Both mechanisms of protection are implicated in spontaneous resolution of HCV infection in humans^20^, ^21^, ^22^ and chimpanzees.^23^, ^24^ All strategies face difficulties dealing with the enormous genetic diversity of HCV,^3^, ^25^ leading to a recent focus targeting conserved HCV genomic regions.^26^ Further questions on vaccine efficacy cannot be answered in chimpanzees because of a recent moratorium on using chimpanzees in biomedical research.^27^

For these reasons, development of a small animal model is critically important for HCV vaccine development. Such a model has been developed in inbred Lewis rats using a human adenoviral vector.^7^ Here, we develop the model in outbred Sprague‐Dawley (SD) rats, more closely mirroring human diversity, with a more clinically relevant chimpanzee adenovirus. We believe the SD rat RHV model will be highly beneficial to the HCV vaccine field in refining optimal antigens, delivery platforms, adjuvants, and prime/boost regimes, all of which can help inform difficult and expensive clinical trials before they begin. The model is also suited to dissecting mechanisms of protection, questions difficult, if not impossible, to answer in humans.

In the present study we examined the protective efficacy of a simian adenovirus encoding nonstructural or structural antigens against RHV challenge in outbred rats. The utility of the model is demonstrated by finding that a T‐cell response focused on nonstructural antigens is partially protective and can be greatly enhanced by boosting either with a second dose of adenovirus or a plasmid encoding the same antigens or with a higher single dose of adenovirus. The efficacy of vaccination using structural antigens alone and in combination with nonstructural antigens is also explored for RHV. Importantly, our results show that vaccination resulting in aviremic protection is possible against an HCV‐like hepacivirus.

## Materials and Methods

### Ethics Statement

All animals and procedures were used in accordance with the terms of the UK Home Office Animals Act Project Licence and approved by the University of Oxford Animal Care and Ethical Review Committee (PPL 30/3293 and P874AC0F0).

### Rat Strains

Outbred male SD rats (Charles River, UK), 225‐250 g at the start of the experiment, were kept under conventional conditions in individually ventilated cages and fed a normal chow diet.

### Infection of Rats

Serum from one rat infected with RHV‐rn1 (accession no. KX905133) was used as the inoculum for all challenge experiments. Serum was diluted to 10^5^ viral particles (vp) of RHV in 100 µL phosphate‐buffered saline (PBS) per dose, unless otherwise stated, administered into the tail vein. Titration of RHV to determine the 100% minimum infectious dose (MID) was performed by 1:10 dilution series from 10^6^ vp.

### Vaccines

An RHV immunogen, coding for the nonstructural 3 (NS3) to NS5 region of RHV‐rn1 with GDD to AAG NS5B mutation, was synthesized using *Mus musculus* codons (GeneArt; ThermoFisher). The construct included a Kozak sequence, truncated shark invariant chain, and V5 epitope tag. SIi‐RHV‐NS3‐NS5_mut_‐V5 was cloned into a pENTR4 vector downstream of the human cytomegalovirus immediate early promoter and tetracycline operator. The coding cassette was moved to the ChAdOx1 destination vector using Thermo Fisher LR gateway cloning, and all steps were checked by sequencing. ChAdOx1 plasmids incorporating SIi‐RHV‐NS3‐NS5_mut_‐V5 linearized with PmeI were transfected into T‐REx‐293 cells (Thermo Fisher) for generation of viral‐vector vaccines. ChAdOx1‐SIi‐RHV‐NS3‐NS5_mut_‐V5 (ChAd‐NS) vaccines were manufactured by the Viral Vector Core Facility (Jenner Institute, University of Oxford). An identical process was followed for production of a ChAdOx1 vector encoding RHV antigen core, E1, E2, p7, and NS2 (ChAd‐S).

### Vaccination

Rats were vaccinated by intramuscular injection into the right hind thigh muscle using 50 µL viral vector or DNA plasmid in PBS. Typically, 10^8^ infectious units (i.u.) of adenovirus vaccine or 100 µg DNA plasmid were administered per dose, unless otherwise stated. A second vaccine when administered was done 11 weeks following the first vaccination (Table 1).

**Table 1:** Vaccination and Challenge Regimes

Group	Initial Vaccination (Dose)	Boost Vaccination (Dose)	Challenge Dose (vp of RHV)
ChAd‐NS (1E6 challenge)	ChAd‐NS (1 × 10^8^ i.u.)	‐	1 × 10^6^ vp
ChAd‐NS (1E5 challenge)	ChAd‐NS (1 × 10^8^ i.u.)	‐	1 × 10^5^ vp
ChAd‐NS (single high dose)	ChAd‐NS (6.8 × 10^8^ i.u.)	‐	1 × 10^5^ vp
ChAd‐NS/ChAd‐NS	ChAd‐NS (1 × 10^8^ i.u.)	ChAd‐NS (1 × 10^8^ i.u.)	1 × 10^5^ vp
ChAd‐NS/DNA	ChAd‐NS (1 × 10^8^ i.u.)	DNA (100 µg)	1 × 10^5^ vp
Mock vaccination	ChAd‐GFP (1 × 10^8^ i.u.)	‐	10^5^ or 10^6^ vp

### Isolation of Lymphocytes

Peripheral blood mononuclear cells (PBMCs) were isolated from tail vein blood using a Vacutainer (Fisher Scientific). Blood was centrifuged (5,000 rpm), the pellet was resuspended in 1 mL ammonium–chloride–potassium (ACK) lysing buffer (Life Technologies; 3‐5 min at room temperature [RT]), diluted in 10 mL PBS, and centrifuged (5 min, 1,800 rpm). The pellet was resuspended in R10: Roswell Park Memorial Institute (Sigma), 10% fetal bovine serum (Sigma), 1% L‐glutamine (Sigma), and 1% penicillin/streptomycin (Sigma).

Splenocytes were isolated from rat spleens by disruption through a 70‐µm cell strainer and centrifuged (1,200 rpm, 5 min, 4°C), and the pellet was resuspended in 3 mL ACK lysing buffer (2 minutes, RT). PBS was added to 25 mL, centrifugation was performed as before, and the pellet was resuspended in R10.

Liver‐infiltrating lymphocytes were obtained by perfusion of the liver with PBS and disruption through a 100‐µm then a 50‐µm cell strainer. Cells were centrifuged (5 minutes, 1,500 rpm), and the pellet was resuspended in 40% Percoll (GE). An underlay of 70% Percoll was applied, and samples were centrifuged (2,000 rpm, 25 minutes without brake). Fat and debris were removed and interphase was collected, diluted in PBS, and centrifuged (1,500 rpm, 5 minutes). The pellet was resuspended in 3 mL ACK and treated as for splenocytes.

### Flow Cytometry

Cells were stimulated overnight with pools of 15–amino acid peptides overlapping by 11, spanning the whole RHV polypeptide sequence (1 µg/mL). Dimethyl sulfoxide (DMSO) was applied to negative controls and phorbol 12‐myristate 13‐acetate/ionomycin to positive controls. Mouse antirat cluster of differentiation 28 (CD28; BD Pharmingen, 1:62.5) was added to each well. One million to 3 million PBMCs per well were incubated with peptide pools or controls (2 hours, 37°C) then Golgi Plug (BD Pharmingen; 1:1,000, 16‐18 hours, 37°C). After incubation with mouse antirat CD32 (BD Pharmingen; 1:100, RT, 15 minutes), a surface‐staining cocktail was applied (30 minutes) (Supporting Table S3), followed by fixation and permeabilization (Cytofix/Cytoperm; BD Biosciences), cell washing (Perm/Wash buffer; BD Biosciences), and intracellular staining (30 minutes) (Supporting Table S1). Cells were acquired on an LSR II cytometer, and the results were analyzed using FlowJo software (version 10).

### Enzyme‐Linked Immunosorbent Spot

MAIP enzyme‐linked immunosorbent spot (ELISpot) plates (Millipore) were coated overnight with 10 µg/mL antirat interferon gamma (IFNγ; Mabtech). Frozen splenocytes were thawed and stimulated in R10 with pools of peptides, individual peptides (4 μg/mL), or DMSO‐negative control. Following incubation (37°C, 18‐20 hours), plates were washed in PBS and treated with antirat IFNγ development reagents per the manufacturer’s instructions (Mabtech). Results were expressed as background‐subtracted spot‐forming units per million splenocytes.

### Serum RNA Extraction

Serum was collected in a Microvette (Sarstedt) from the tail vein of rats. RNA from serum samples was extracted using the QIAamp Viral RNA Mini Kit per the manufacturer’s instructions.

### Quantitative PCR

Quantitative PCR to determine genome equivalents of RHV per milliliter of serum was performed as described,^6^ using a Quantifast SYBR Greeen RT‐PCR kit (Qiagen), with copy numbers determined against a standard curve of full‐length RHV RNA.

### Next‐Generation Sequencing

RNA was extracted from blood samples using a viral RNA mini kit (Qiagen). RNA was subjected to complementary DNA and second‐strand DNA synthesis using SuperScript III reverse transcriptase (Invitrogen) and Sequenase, version 2.0 (Thermo Fisher). Libraries were prepared from DNA using Nextera XT kit (Illumina) and sequenced on the MiSeq platform with the reagent kit (500 cycles). After adapter removal and quality trimming, reads were aligned against the reference sequence (Genbank no. KX905133) using Bowtie2, and variant calling was performed using GATK with HaplotypeCaller.

### Statistics

Statistical tests for association of immune responses with challenge outcome were performed using the Kruskal‐Wallis test with Dunn's test correcting for multiple comparisons. Comparison of final viremic status for each vaccination regime with mock‐vaccinated rats was performed using Fisher's exact test.

## Results

### RHV MID

**Figure 1:**
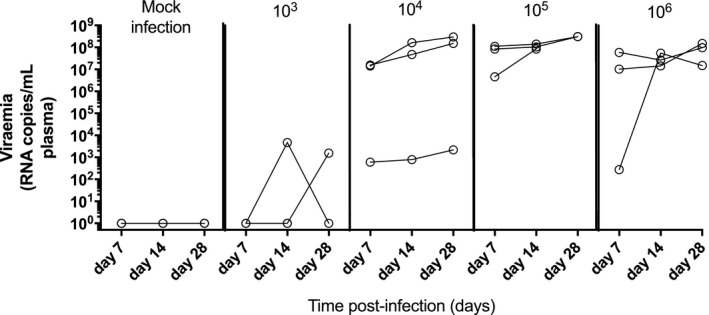
Determination of MID of RHV. Male SD rats (n = 3/group) were administered varying doses of RHV (10^3^, 10^4^, 10^5^, or 10^6^ vp) by intravenous injection into the tail vein. Viremia was determined from blood sampling 7, 14, and 28 days postinfection by quantitative PCR and expressed as RHV genome equivalents per millilitre of plasma.

To determine the MID of RHV, the inoculum was titrated by 10‐fold dilution and administered to SD rats (n = 3/group) (Fig. 1). Mock infection produced no detectable viremia by quantitative PCR. A dose of 1 × 10^3^ RNA genome copies (vp) of RHV resulted in two thirds of rats infected. Doses between 1 × 10^4^ and 1 × 10^6^ vp resulted in 100% infection. Unless indicated otherwise, 10 × MID at 1 × 10^5^ vp of RHV was used for challenge studies.

### Immune Responses to RHV Infection in Unvaccinated Rats

**Figure 2:**
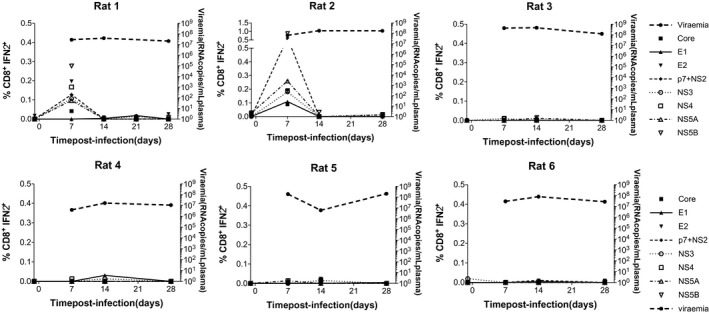
Blood CD8^+^IFNγ^+^ immune responses to RHV infection in unvaccinated rats. Six male SD rats were infected with 10^6^ vp of RHV by intravenous injection into the tail vein. PBMCs were isolated and stimulated with eight pools of overlapping 15‐mer peptides spanning the entire polypeptide sequence of RHV. Blood samples were taken weekly for 4 weeks postinfection to chart a time course of blood CD8^+^IFNγ^+^ responses by flow cytometry.

The cellular immune response was quantified in six unvaccinated SD rats infected with 10^6^ vp. A time course of blood CD4^+^IFNγ^+^ (Supporting Fig. S1) and CD8^+^IFNγ^+^ (Fig. 2) responses 4 weeks postinfection revealed minimal response to any peptide pools spanning the RHV polyprotein. The only exception was a high but transient CD8^+^IFNγ^+^ response in two rats 7 days postinfection, with no discernible effect on viremia. Splenocyte (Supporting Fig. S2A,B) and liver‐infiltrating lymphocyte (Supporting Fig. S2C,D) responses were minimal.

### Immunogenicity of Adenovirus Vector Vaccine Encoding Nonstructural Proteins of RHV

**Figure 3:**
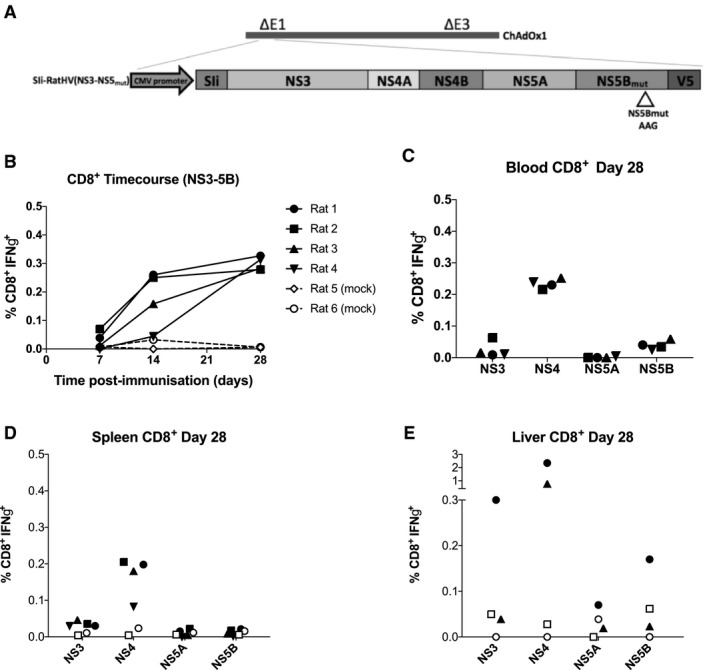
CD8^+^IFNγ^+^ cellular immune responses following vaccination of rats with an adenoviral vectored vaccine encoding the nonstructural proteins of RHV (ChAd‐NS). Four male SD rats were vaccinated intramuscularly with 10^8^ i.u. per dose of an adenoviral (ChAdOx1) vectored vaccine encoding the nonstructural proteins of RHV shown in (A). Truncated shark invariant chain was used as a genetic adjuvant. PBMCs were isolated from blood drawn weekly from vaccinated rats and stimulated with four pools of overlapping peptides representing the nonstructural regions of RHV. (B) The time course of CD8^+^IFNγ^+^ responses for 4 weeks postvaccination, as measured using flow cytometry; plotted are pooled responses against all four peptide pools for each rat. Mock‐vaccinated rats are shown with hatched lines. (C‐E) Immune responses by flow cytometry 4 weeks postvaccination against the four individual peptide pools as shown for PBMCs, splenocytes, and liver‐infiltrating lymphocytes, respectively, with mock‐vaccinated rats shown as unfilled symbols. Abbreviations: CMV, cytomegalovirus; SIi, shark invariant chain.

A simian adenovirus (ChAdOx1) vector vaccine was constructed encoding nonstructural proteins of RHV linked to a truncated shark invariant chain known to enhance T‐cell responses (ChAd‐NS) (Fig. 3A).^28^ A time course of CD4^+^IFNγ^+^ (Supporting Fig. S3A) and CD8^+^IFNγ^+^ (Fig. 3B) PBMC responses revealed little or no CD4^+^IFNγ^+^ response, but a CD8^+^IFNγ^+^ response focused on NS4 peaked 4 weeks after vaccination with a mean of 0.3% NS‐specific CD8^+^IFNγ^+^ T cells (Fig. 3C). These results were similar in splenocytes (Fig. 3D; Supporting Fig. S3B) and liver‐infiltrating lymphocytes (Fig. 3E; Supporting Fig. S3C).

### Epitope Mapping of Immunodominant Peptides in Infected and Vaccinated Rats

Three RHV‐infected rats showed minimal response to any 15‐mer peptide spanning the RHV polyprotein except one from the core region (Supporting Fig. S4A). In a vaccinated rat one peptide from the NS3 region and two from NS4 (Supporting Table S1) elicited strong CD8^+^ responses (Supporting Fig. S4B,C).

### Protective Efficacy of ChAd‐NS Vaccine Against RHV Challenge

**Figure 4:**
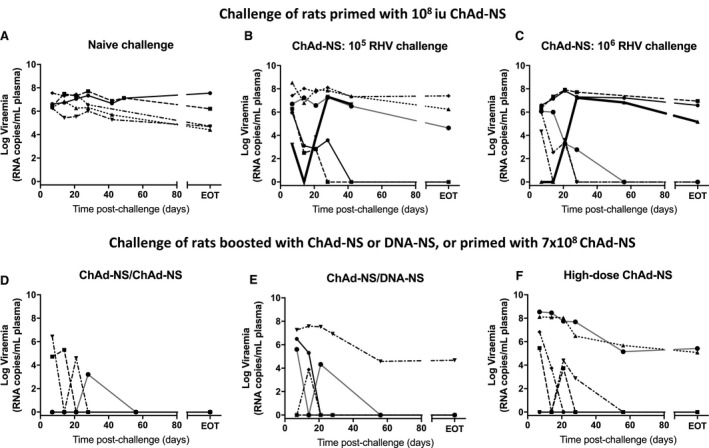
Protective efficacy of ChAd‐NS vaccination strategies against challenge with RHV. Male SD rats (n = 6/group) were vaccinated and challenged as described in Table 1. Vaccinations were administered by intramuscular injection into the right hind leg. Four weeks following vaccination RHV was administered by intravenous injection into a tail vein. (A) Mock‐vaccinated rats, challenged with either 10^5^ or 10^6^ vp RHV. (B‐F) Rats vaccinated and challenged as indicated. Viremia at each time point obtained by quantitative PCR against a standard curve of RHV genomes of known concentration.

To test the efficacy of ChAd‐NS vaccination, SD rats (n = 6) were vaccinated with 1 × 10^8^ i.u. and challenged 4 weeks later with 1 × 10^5^ vp (Supporting Fig. S5A). All mock‐vaccinated rats rapidly developed high viremia, sustained until end of trial (EOT; approximately 150 days postinfection) (Fig. 4A). All rats vaccinated with ChAd‐NS became infected, but two of six showed no detectable viremia in blood by 6‐7 weeks postinfection, a status unchanged by EOT (Fig. 4B). Increasing the challenge dose to 1 × 10^6^ vp showed little effect on efficacy: three of six rats cleared infection 6‐7 weeks later and remained clear until EOT (Fig. 4C). This equates to a combined efficacy of 42% using 1 × 10^8^ i.u. ChAd‐NS (Supporting Fig. S5C).

To determine whether additional vaccination strategies could improve efficacy, two groups of six SD rats were primed with 1 × 10^8^ i.u. ChAd‐NS and boosted 11 weeks later with a second dose of 1 × 10^8^ i.u. ChAd‐NS (Fig. 4D) or 100 µg DNA plasmid encoding the same antigens (Fig. 4E). A third group of rats (n = 6) was administered a single 7‐fold higher ChAd‐NS vaccine dose of 6.8 × 10^8^ i.u. (Fig. 4F). All groups were challenged 4 weeks after final vaccination with 1 × 10^5^ vp (Supporting Fig. S5A; Table 1). These groups displayed substantially greater protection than rats receiving a single dose of 1 × 10^8^ i.u. ChAd‐NS, at both day 28 and EOT (Supporting Fig. S5B,C). For the ChAd‐NS boost, DNA boost, and single high‐dose groups, efficacy was 100% (*P* = 0.001), 83% (*P* = 0.008), and 67% (*P* = 0.03), respectively (Supporting Fig. S5C).

Six rats out of the 18 forming these latter three groups showed no evidence of viremia (aviremia) at any time point, consistent with “sterilizing” immunity. Aviremia was observed in the ChAd‐NS boost, DNA boost, and high‐dose ChAd‐NS groups at a frequency of 50%, 33%, and 17%, respectively (Supporting Fig. S5C), but was not observed for any rat receiving a single dose of 1 × 10^8^ i.u. ChAd‐NS (Fig. 4B,C).

### Immune Responses in Vaccinated, Challenged Rats and Association with Protection

**Figure 5:**
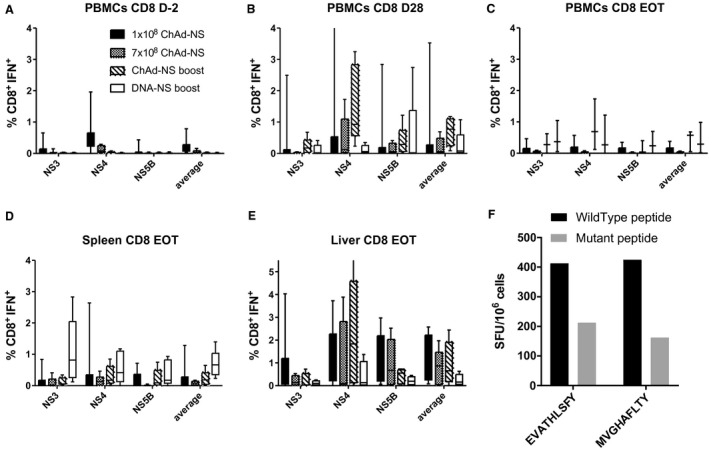
Effects on CD8^+^IFNγ^+^ cellular immunogenicity of different vaccination regimes and putative escape mutation. Male SD rats (n = 6/group) were vaccinated and challenged 4 weeks after final vaccination as described in Table 1. CD8^+^IFNγ^+^ responses from PBMCs are shown (A) 2 days prior to infection, (B) 4 weeks postinfection, and (C) at EOT. CD8^+^IFNγ^+^ responses at EOT are shown for (D) splenocytes and (E) liver‐infiltrating lymphocytes. (F) A rat vaccinated by intramuscular injection of 10^8^ i.u. ChAd‐NS and challenged 4 weeks later by intravenous injection of 10^6^ vp of RHV showed initial control of virus but subsequent breakthrough infection. RHV RNA was isolated from serum taken following breakthrough and sequence data obtained by next generation sequencing. Shown are the ELISpot responses from splenocytes stimulated with either wild‐type (black bars) or mutant (gray bars) forms of putative T‐cell epitopes (epitopes listed as wild‐type sequences). Abbreviation: SFU, spot‐forming unit.

**Figure 6:**
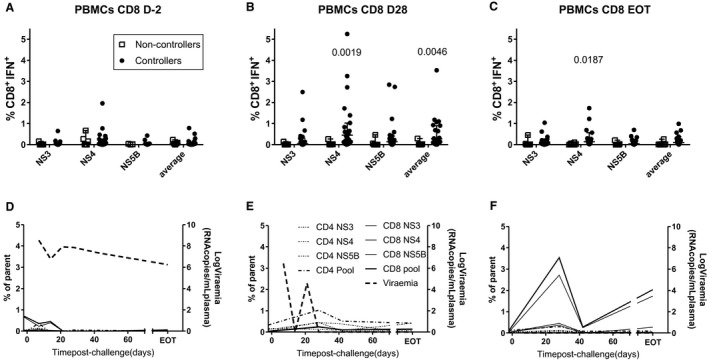
Association of CD8^+^IFNγ^+^ cellular immune responses with protection. Male SD rats (n = 6/group) were vaccinated and challenged 4 weeks after final vaccination as described in Table 1. Rats were divided into two groups, "controllers" and "noncontrollers," on the basis of possessing detectable viremia or otherwise at EOT. Associations of CD8^+^IFNγ^+^ responses from PBMCs are shown (A) 2 days prior to infection, (B) 4 weeks postinfection, and (C) at EOT. Numbers on graphs represent significant *P* values between indicated groups using analysis of variance with Bonferroni's multiple comparison test. Representative time courses of cellular responses and viremia in three vaccinated rats which (D) did not control infection, (E) resolved an infection, or (F) showed no detectable infection.

To determine whether differences in cellular responses might be responsible for different challenge outcomes, immune responses prechallenge and postchallenge and their association with protection were compared between groups (Figs. 5 and 6; Supporting Figs. S6 and S7). Two marked differences were observed between rats vaccinated with the standard dose of 1 × 10^8^ i.u. and those boosted with ChAd‐NS or DNA‐NS or administered a higher single dose of ChAd‐NS. The latter three groups showed higher recall of CD4 (Supporting Fig. S6B) and CD8 (Fig. 5B) responses 28 days postinfection, particularly against NS4. There were no clear differences in cellular immune responses between vaccine groups prior to challenge (Supporting Fig. S6A; Fig. 5A) or at EOT (Supporting Fig. S6C,D; Fig. 5C‐E). The population of liver‐infiltrating CD8^+^ lymphocytes remained at high levels until EOT in all vaccinated groups (Fig. 5E).

Cellular responses varied markedly with outcome (Fig. 6D‐F; Supporting Figs. S8 and S9). To test for statistically significant associations between cellular immune responses and infection resolution, vaccinated rats were divided into two groups: “controllers” with undetectable viremia and “noncontrollers” with high viremia, at EOT (Supporting Fig. S5B). Two rats showing early control but later breakthrough infection were classified as controllers. CD4^+^IFNγ^+^ (Supporting Fig. S7) and CD8^+^IFNγ^+^ (Fig. 6) responses were higher in controllers than noncontrollers. This association was statistically significant only with CD8^+^IFNγ^+^ responses against NS4 postchallenge (Fig. 6B,C; Supporting Fig. S7E,F). Splenocyte, but not liver‐infiltrating lymphocyte, CD8^+^IFNγ^+^ responses correlated with PBMC CD8^+^IFNγ^+^ responses (Supporting Fig. S7D), suggesting that responses in the liver are not representative of those measured in the blood, providing one possible explanation for the lack of correlation between prechallenge PBMCs and challenge outcome.

### T‐Cell Escape Mutations

Two rats vaccinated with ChAd‐NS showed initial control of RHV infection but subsequent breakthrough infection (Fig. 5B,C, thick lines). To determine whether this was caused by T‐cell escape mutants, the RHV genome sequence from one breakthrough rat was isolated (Fig. 5C) and compared to RHV sequences from two nonvaccinated rats. Seventeen–amino acid substitutions were discovered across 14 putative nonamer T‐cell epitopes (SYFPEITHI software prediction) (Supporting Table S2). The vaccinated breakthrough rat had all but one of these mutations, with the RHV‐infected unvaccinated rats showing mutations in five to seven sites. Two of the mutated nonamers derived from 15‐mer sequences identified as immunodominant in vaccinated rats (Supporting Table S2 and Fig. S4B,C). These nonamers as wild‐type were immunogenic by ELISpot, whereas responses against the mutant forms were lower in the breakthrough rat (Fig. 5F). Interestingly, the epitope MVGHAFLTY from NS4B, associated with resolution of infection (Fig. 6), contained three mutated sites in the RHV isolated from the breakthrough rat. Two of these mutated sites, the underlined valine and alanine residues, were predicted to serve as anchor residues for contiguous nonamer epitopes within the 15‐mer peptide.

### Immunogenicity and Efficacy of Adenovirus Encoding RHV Structural Antigens (ChAd‐S) Alone and in Combination with ChAd‐NS

**Figure 7:**
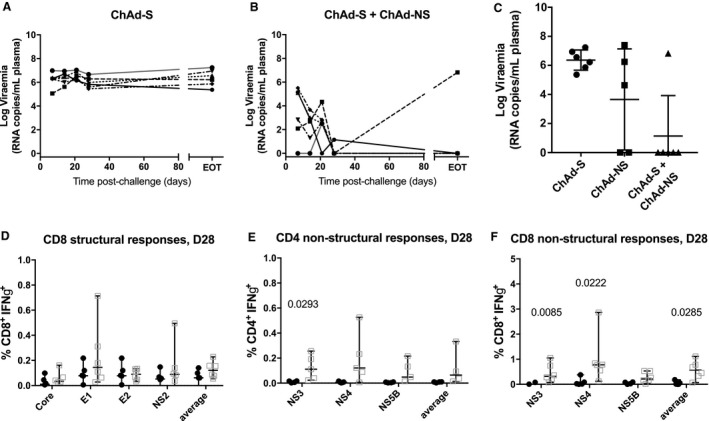
Protective efficacy of ChAd‐S alone and in combination with ChAd‐NS. Male SD rats (n = 6/group) were vaccinated by intramuscular injection with either ChAd‐S, ChAd‐NS, or premixed ChAd‐S and ChAd‐NS at a dose of 10^8^ i.u. per distinct vaccine. Four weeks following vaccination, rats were challenged by intravenous injection into the tail vein of 10^5^ vp RHV. Blood viremia was traced weekly postinfection by real‐time quantitative PCR against a standard curve of RHV RNA for (A) ChAd‐S‐vaccinated and (B) ChAd‐S + ChAd‐NS–vaccinated rats. (C) Viremia at EOT. Blood cellular immune responses were compared by flow cytometry for single‐vaccine versus combination groups for (D) CD8^+^IFNγ^+^ responses to structural antigens and (E) CD4^+^IFNγ^+^ and (F) CD8^+^IFNγ^+^ responses to nonstructural antigens at 28 days postchallenge.

To investigate the role of RHV antigen target in protection, a ChAdOx1‐based vaccine encoding structural antigens, ChAd‐S, was used to vaccinate six SD rats at 1 × 10^8^ i.u. per dose. A second set of rats (n = 6) were vaccinated with a combination of ChAd‐S+ChAd‐NS, with each distinct vaccine used at 1 × 10^8^ i.u. within the mixture. Four weeks postvaccination rats were challenged with 1 × 10^5^ vp. ChAd‐S‐vaccinated rats showed no evidence of viral control postinfection (Fig. 7A). However, the combination of ChAd‐S+ChAd‐NS showed superior viral control at all time points compared against ChAd‐S alone and ChAd‐NS alone (Fig. 7B): 83% of ChAd‐S+ChAd‐NS‐vaccinated rats cleared infection by EOT, compared to 0% for ChAd‐S‐vaccinated and 50% for ChAd‐NS‐vaccinated rats (Fig. 7C).

Analysis of cellular immune responses revealed no difference between single or combination vaccination groups in CD4^+^IFNγ^+^ or CD8^+^IFNγ^+^ responses prior to infection, for any structural or nonstructural antigen (Supporting Figs. S10A,D and S11A,D). CD8^+^IFNγ^+^ responses to structural antigens prior to challenge were focused on E1 and E2 (Supporting Fig. S11D) but broadened to include core and NS2 postchallenge (Fig. 7D). CD4^+^IFNγ^+^ responses to NS3 and CD8^+^IFNγ^+^ responses to NS3 and NS4 were significantly augmented compared to ChAd‐NS‐vaccinated rats 28 days postinfection (Fig. 7E,F). The improved efficacy seen with the ChAd‐S+ChAd‐NS combination compared to single‐vaccine groups may result from an augmented response to NS4, a broadening of the immune response to include structural antigens, or both.

## Discussion

RHV infection of SD rats has enabled a comprehensive evaluation of the efficacy of several different vaccine strategies within a small animal HCV model. Using a homologous prime‐boost approach with a simian adenovirus vaccine encoding nonstructural antigens, 100% resolution of infection was obtained. This study also established that a cellular immune response can prevent any detectable infection by a hepacivirus postchallenge in a vaccinated animal. In summary, the current study establishes the utility of the RHV SD challenge model for rapid and inexpensive assessment of vaccine regimes congruous with current ethical scientific guidelines. The model will be crucial in developing the next‐generation HCV vaccine, work urgently required given the failure of the first clinical trial of HCV vaccine efficacy (NCT01436357).^10^ In that trial, viral‐vectored delivery of nonstructural HCV antigens showed no efficacy. Here, we have shown that the addition of structural to nonstructural antigens markedly enhances efficacy. Other strategies can be explored using the RHV rat model, but most crucially it will be of use in gaining an understanding of the mechanisms of vaccine‐induced protective immunity, enabling rational selection of the next‐generation HCV vaccine.

Similarly to inbred Lewis rats,^7^ RHV infection of unvaccinated SD rats elicited little detectable T‐cell response. In humans a lack of CD4^+^ response during acute infection is associated with development of HCV chronicity.^29^, ^30^ Consistent with this, all naive SD rats infected with RHV in this study maintained high viremia for approximately 150 days, with no spontaneous resolution. The absence of reactive CD8^+^ T cells against RHV contrasts with detectable levels in nearly all humans acutely infected with HCV^31^; their being largely absent in RHV‐infected rats suggests that RHV may possess some additional mechanism of control of host cellular response or that the high levels of early viral replication exacerbate the tolerance mechanisms associated with liver presentation, seen in high‐dose adenovirus models.^32^ Given the lack of immunogenicity of RHV challenge in naive rats and the lack of spontaneous control in this model compared to human infection with HCV, the protection data shown here are encouraging that this vaccine approach could provide robust protection in a clinical setting.

Vaccination with ChAd‐NS induced a strong CD8^+^IFNγ^+^ response, focused on NS4, but no detectable CD4^+^IFNγ^+^ response. In other studies, adenoviruses delivering HCV NS proteins show a similar skew toward CD8^+^ T lymphocytes in humans,^3^, ^33^, ^34^ macaques,^35^, ^36^ and Lewis rats.^7^ Similar to our findings, boosting with a second adenovirus did little to increase CD8^+^ or CD4^+^ T‐lymphocyte responses in humans, but an MVA boost did markedly increase both^34^; it will be of interest to determine whether an adenovirus/MVA prime‐boost regime demonstrates the same profile of immunogenicity in rats and whether this results in enhanced protection against RHV challenge.

The efficacy (42%) of ChAd‐NS against RHV in SD rats was of the same magnitude seen using a 5‐fold higher dose of adenovirus serotype 5 (HuAd5) to deliver NS antigens in Lewis rats (66%).^7^ The CD4^+^ response has been established as important for control of HCV in chimpanzees,^37^ humans,^29^, ^30^ and Lewis rats.^7^ It will be of interest to see if CD4^+^ T cells play the same role in the protection against RHV in SD rats; no evidence of an association of CD4^+^IFNγ^+^ responses with protection was found here. Another potential difference between the two models is the specific role of the adenovirus backbone in driving protective immunity. HuAd5 has limited capacity for translation due to widespread prior immunity,^38^ while ChAdOx1 is associated with higher levels of innate activation with strong translational potential due to lack of preexisting antibody.^39^, ^40^ The exact role of innate activation in the adjuvanticity of adenoviral vector biology is still not fully understood but can impact on immunogenicity and therefore protective capacity.^41^, ^42^

Boosting with ChAd‐NS or DNA‐NS or using a 7‐fold higher single dose enhanced resolution compared to ChAd‐NS at the standard priming dose. One third of these rats (6/18) showed no detectable RHV viremia at any time point, an outcome not seen with the standard dose of ChAd‐NS in SD or Lewis rats.^7^ This result demonstrates that a suboptimal vaccination regime against a hepacivirus can be transformed into a highly protective regime through boosting or a higher single dose, suggesting that in the case of clinical trials of HCV vaccines resulting in low but statistically significant efficacy, large improvements could be achievable with minor adjustments in the vaccination regimen, warranting such further clinical testing.

The improved efficacy of the boost or higher prime‐dose regimes was associated with a stronger CD8^+^IFNγ^+^ recall response in blood, spleen, and liver but not with blood cellular responses prior to challenge. The inability of prechallenge blood cellular responses to predict postchallenge outcome suggests that some aspect of immunity not measured here may be a better preinfection vaccine correlate of protection, such as memory,^18^, ^37^, ^43^ liver‐residence^43^ polyfunctionality, or granzyme expression. It is worth noting that we observed no correlation between liver‐infiltrating lymphocytes and PBMC responses at EOT, and in a study in vaccinated Lewis rats RHV‐specific CD4^+^ T cells were detectable only in the liver postchallenge, not in the spleen.^7^ In one chimpanzee vaccine study, vaccine‐induced CD8^+^ alone did not associate with protection, but CD8^+^ markers of activation did.^19^ In another chimpanzee vaccine study, proinflammatory cytokines in the liver did not, whereas markers of tolerance did, associate with challenge outcome.^44^ These studies demonstrate the potential complexities in defining a reliable prechallenge correlate of protection.^45^ One of the most practical applications of the rat RHV model will be to determine a reliable vaccine correlate of protection that can apply to clinical trials of HCV vaccines, which, if validated in humans, would enhance progress in HCV vaccine development by obviating the need for challenge studies for each vaccine. Further mechanistic work will also be required to explain cases of aviremia, with available data not suggesting a clear role for either cellular or humoral responses.

A well‐defined feature of HCV infection is CD8^+^ T‐cell escape mutants contributing to persistence,^37^, ^46^ also observed in Lewis rats.^7^ Mutations were found concurrently in both the immunodominant CD8^+^ T‐cell epitopes defined in the present study; given that SD rats are outbred and the major histocompatibility complexes were not haplotyped, this is suggestive of escape mutation but not conclusive. It is not known whether T‐cell escape mutations can be prevented with a sufficiently strong immune response to vaccination; interestingly, in this study, the two rats wherein escape mutants arose were administered the suboptimal single‐prime dose of ChAd‐NS (2/12 rats), whereas breakthrough infections were not observed in any of the rats vaccinated with the more protective boost or high‐priming dose regimes. Two further questions could be answered, exploiting viral escape in the rat RHV model. Firstly, an escape mutant can allow heterologous challenge to be approximated in the rat RHV model. Secondly, a vaccine missing the immunodominant epitopes could be constructed to test the hypothesis that its absence will increase the cellular immune response to subdominant epitopes^47^ and whether this strategy enhances efficacy.

The strategy to broaden the immune response to both structural and nonstructural antigens by combining ChAd‐NS with ChAd‐S clearly demonstrated enhanced efficacy compared to either vaccine alone. The exact mechanism for enhanced protection is unclear as alone ChAd‐S confers no efficacy. Nevertheless, this combination shows the highest‐level efficacy of any single‐shot vaccination regimen tested in rats. It is remarkable that the addition of a vaccine which shows no protection alone has such a profound impact in this challenge model. Given the clear role for immune escape seen in vaccine failure, one explanation could be driving a critical threshold of breadth, seen as critical in many T‐cell vaccine strategies.^48^ Because these antigens were delivered using separate adenoviruses, this may also have served to allow for maximization of breadth, as recently observed using adenovirus vectors in murine studies.^49^, ^50^ Antibodies raised by the structural antigens may also play a role in protection; passive antibody transfer to naive and ChAd‐NS‐vaccinated rats will determine this more fully.

Further work using this model can now proceed to investigate other vaccination platforms, adjuvants, and antigen combinations, all of which will inform and accelerate clinical vaccine development for HCV. The extremely high levels of protection obtained both by prime‐boost regimes and by a single‐shot vaccination strategy combining structural and nonstructural antigens warrant further mechanistic investigation. Such investigation would be of great utility in designing optimal vaccines for humans; the ease of working with rats will allow a reliable vaccine correlate of protection to be established for high‐level protection against HCV‐like hepaciviruses.
