# Supplementary material for: Use of an Outbred Rat Hepacivirus Challenge Model for Design and Evaluation of Efficacy of Different Immunization Strategies for Hepatitis C Virus
Source: Hepatology. 2019 Oct 11;71(3):794–807. doi: 10.1002/hep.30894 (PMC7154631; doi:10.1002/hep.30894)
Supplement: Supplementary file 1 [file HEP-71-794-s001.pdf]

Supplemental Figure Legends

Supplemental Figure 1: Blood CD4$^+$IFNγ$^+$ immune responses to RHV infection in unvaccinated rats. 6 male Sprague-Dawley rats were infected with 10^6 viral particles of RHV by intravenous injection. PBMCs were isolated and stimulated with 8 pools of overlapping 15mer peptides spanning the entire polypeptide sequence of RHV. Blood samples were taken weekly for four weeks post-infection to chart a time-course of blood CD4$^+$IFNγ$^+$ responses by flow cytometry.

Supplemental Figure 2: Spleen and liver immune responses to RHV infection in unvaccinated rats. 6 male Sprague-Dawley rats were infected with 10^6 viral particles of RHV by intravenous injection. Four weeks post-infection rats were sacrificed; splenocytes were isolated from the spleen (A, B) and liver-infiltrating lymphocytes from the liver (C, D) and stimulated with 8 pools of overlapping peptides spanning the length of the RHV polypeptide. (E) Representative gating strategy for flow cytometry on Rat 1.

Supplemental Figure 3: CD4$^+$IFNγ$^+$ cellular immune responses following vaccination of rats with an adenoviral vectored vaccine encoding the non-structural proteins of RHV (ChAd-NS). Four male Sprague-Dawley rats were vaccinated with 10^8 infectious units per dose of ChAd-NS. PBMCs were isolated from blood drawn weekly from vaccinated rats and stimulated with four pools of peptides representing the non-structural regions of RHV. (A) shows the time-course of CD4$^+$IFNγ$^+$ responses for four weeks post-vaccination, as measured using flow cytometry; plotted are pooled responses against all four peptide pools for each rat. Mock-vaccinated rats are shown with hatched lines. (B) and (C) show the immune responses by flow cytometry four weeks post-vaccination against the four individual peptide pools as

shown, for splenocytes and liver-infiltrating lymphocytes respectively, with mock-vaccinated rats shown as squares.

Supplemental Figure 4: Identification of immunodominant epitopes in infected and vaccinated rats. IFNγ ELISpots followed by flow cytometry were performed on splenocytes from infected (A) and vaccinated (B) rats to identify peptides responsible for $CD8^{+}IFN\gamma^{+}$ responses. Sub-pools of peptides and then individual peptides were screened and responses plotted as spot-forming units (SFU) per million splenocytes (ELISpot) or as % parent (flow cytometry, 4C) as shown.

Supplemental Figure 5: Schema and summary of protective efficacy of ChAd-NS vaccination strategies against challenge with RHV. Male Sprague-Dawley rats (n=6 per group) were vaccinated and challenged as described in Table 1. Vaccinations were administered by intramuscular injection into the right hind leg. Four weeks following vaccination RHV was administered by intravenous injection into a tail vein as shown in (A). Viraemia at each time-point obtained by quantitative PCR against a standard curve of RHV genomes of known concentration. Viraemia at (B) 28 days post-challenge and (C) at end of trial (approx. 150 days post-infection). (D) Summary of protective efficacy and aviraemia seen in each vaccination group. “ChAd-NS” represents combined results from groups (n=6 each) challenged with 10^5 and 10^6 vp RHV respectively.

Supplemental Figure 6: Effects on $CD4^{+}IFN\gamma^{+}$ cellular immunogenicity of different vaccination regimes. Male Sprague-Dawley rats (n=6 per group) were vaccinated and challenged four weeks after final vaccination as described in Table 1. $CD4^{+}IFN\gamma^{+}$ responses

from PBMCs are shown (A) two days prior to infection (B) four weeks post-infection and (C) at end of study, and (D) for splenocytes at end of study.

Supplemental Figure 7: Association of $CD4^{+}IFN\gamma^{+}$ and $CD8^{+}IFN\gamma^{+}$ ccellular immune responses with protection. Male Sprague-Dawley rats (n=6 per group) were vaccinated and challenged four weeks after final vaccination as described in Table 1. Rats were divided into two groups, "controllers" and "non-controllers", on the basis of possessing detectable viraemia or otherwise at end of trial. Associations of $CD4^{+}IFN\gamma^{+}$ responses from PBMCs are shown (A) two days prior to infection (B) four weeks post-infection and (C) at end of study. (D) Correlations between $CD8^{+}IFN\gamma^{+}$ liver-infiltrating lymphocyte or splenocyte responses and PBMC responses at EOT. (E) $CD4^{+}IFN\gamma^{+}$ and (F) $CD8^{+}IFN\gamma^{+}$ responses at end of study are shown for splenocytes and (G) liver-infiltrating lymphocytes.

Supplemental Fig 8: Individual time-course of viraemia and PBMC immune responses in ChAd-NS-vaccinated and challenged rats which controlled infection. Male Sprague-Dawley rats were vaccinated and challenged as described in Table 2. Blood cellular immune responses were obtained at each time-point by flow cytometry by stimulation with pools of peptides representing three regions, NS3, NS4 and NS5B. Viraemia was obtained by qPCR against a standard curve of RHV genome copies of known concentration on serum samples at indicated time-points.

Supplemental Fig 9: Individual time-course of viraemia and blood cellular immune responses in ChAd-NS-vaccinated and challenged rats: non-controllers, breakthrough infections, and aviraemic. Male Sprague-Dawley rats were vaccinated and challenged as described in Table 2. Blood cellular immune responses were obtained at each time-point by

flow cytometry by stimulation with pools of peptides representing three regions, NS3, NS4 and NS5B. Viraemia was obtained by qPCR against a standard curve of RHV genome copies of known concentration on serum samples at indicated time-points. (A) Non-controllers; (B) Breakthrough infections; (C) Aviraemic.

Supplemental Figure 10: Blood cellular immunogenicity against structural antigens of ChAd-S alone and in combination with ChAd-NS. Male Sprague-Dawley rats (n=6 per group) were vaccinated by intramuscular injection with either ChAd-S, ChAd-NS or pre-mixed ChAd-S and ChAd-NS at a dose of 10^8 i.u. per distinct vaccine. Four weeks following vaccination rats were challenged by intravenous injection into the tail vein of 10^5 viral particles RHV. Blood cellular immune responses were compared by flow cytometry against structural antigens for single-vaccine versus combination groups for (A-C) $CD4^+IFN\gamma^+$ and (D-E) $CD8^+IFN\gamma^+$ cell types at timepoints indicated.

Supplemental Figure 11: Blood cellular immunogenicity against non-structural antigens of ChAd-S alone and in combination with ChAd-NS. Male Sprague-Dawley rats (n=6 per group) were vaccinated by intramuscular injection with either ChAd-S, ChAd-NS or pre-mixed ChAd-S and ChAd-NS at a dose of 10^8 i.u. per distinct vaccine. Four weeks following vaccination rats were challenged by intravenous injection into the tail vein of 10^5 viral particles RHV. Blood cellular immune responses were compared by flow cytometry against non-structural antigens for single-vaccine versus combination groups for (A-B) $CD4^+IFN\gamma^+$ and (C-D) $CD8^+IFN\gamma^+$ cell types at timepoints indicated. Numbers above comparison groups represent p-values from ANOVA with Bonferroni's multiple comparisons test.

Supplemental Figure 12: Spleen cellular immunogenicity of ChAd-S alone and in combination with ChAd-NS. Male Sprague-Dawley rats (n=6 per group) were vaccinated by intramuscular injection with either ChAd-S, ChAd-NS or pre-mixed ChAd-S and ChAd-NS at a dose of 10^8 i.u. per distinct vaccine. Four weeks following vaccination rats were challenged by intravenous injection into the tail vein of 10^5 viral particles RHV. $CD8^+IFN\gamma^+$ Splenocyte responses were compared by flow cytometry against (A) structural and (B) non-structural antigens for single-vaccine versus combination groups at EOT by flow cytometry.

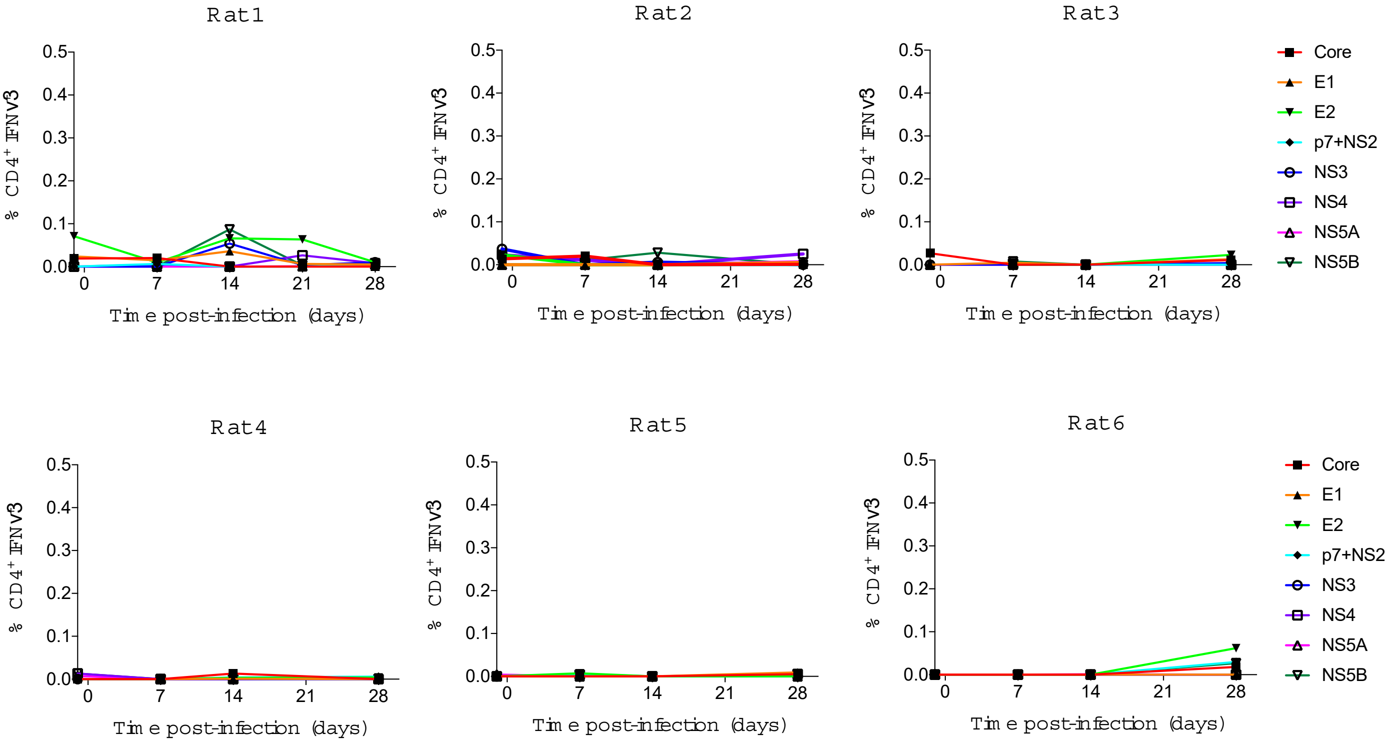

Supplemental Figure 1: Blood CD4$^+$IFNγ$^+$ immune responses to RHV infection in unvaccinated rats
6 male Sprague-Dawley rats were infected with 10^6 viral particles of RHV by intravenous injection. PBMCs were isolated and stimulated with 8 pools of 15mer peptides spanning the entire polypeptide sequence of RHV. Blood samples were taken weekly for four weeks post-infection to chart a time-course of blood CD4$^+$IFNγ$^+$ responses by flow cytometry.

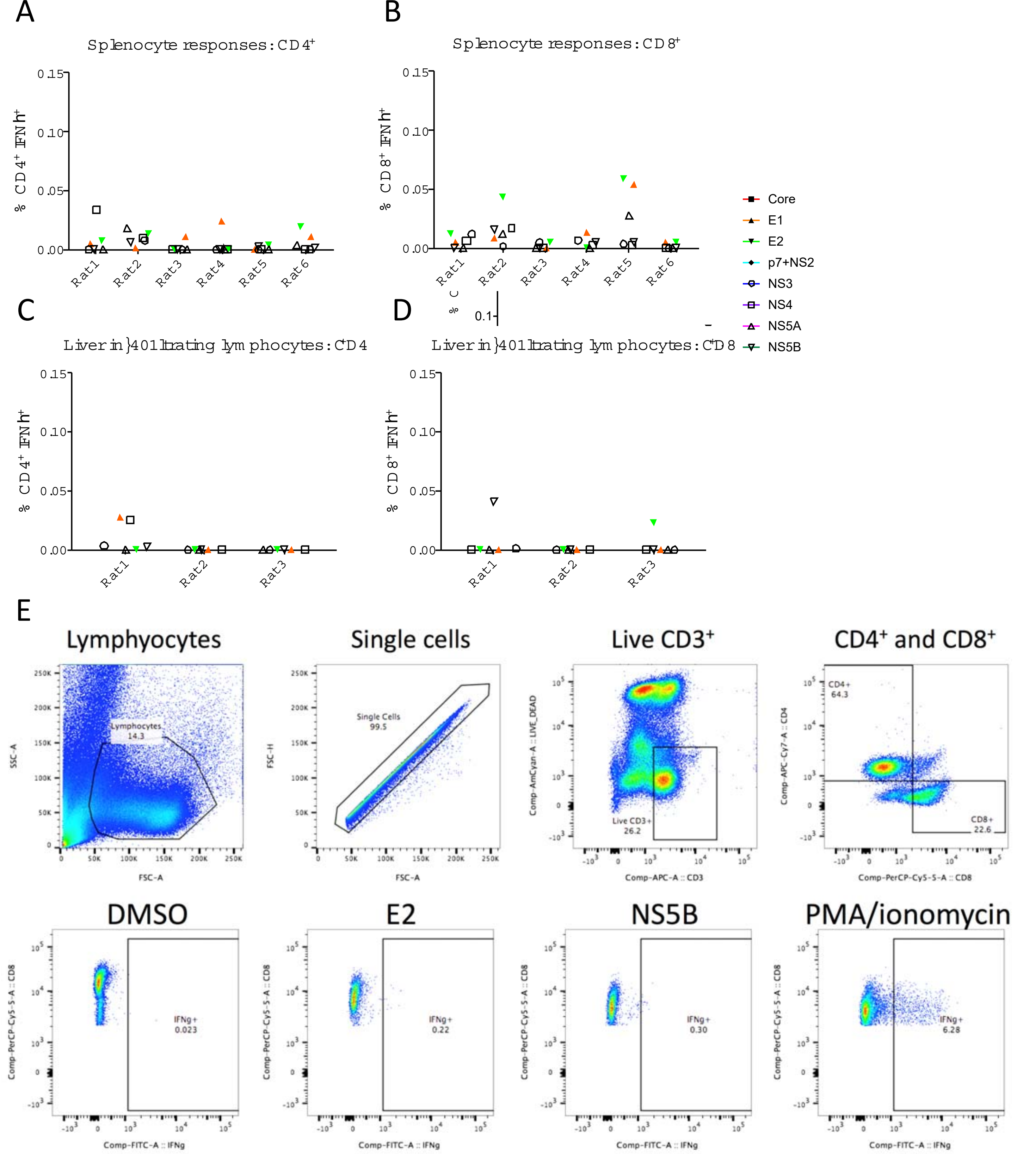

Supplemental Figure 2: Spleen and liver immune responses to RHV infecŸon in unvaccinated rats

6 male Sprague-Dawley rats were infected with 10^6 viral parŸcles of RHV by intravenous injecŸon. Four weeks post-infecŸon rats were sacri}401ced; splenocytes were isolated from the spleen (A, B) and liver in}401ltrating lymphocytes from the liver (C, D) and sŸmulated with 8 pools of pepŸdes spanning the length of the RHV polypepŸde. (E) RepresentaŸve gaŸng strategy for }402ow cytometry on Rat 1.

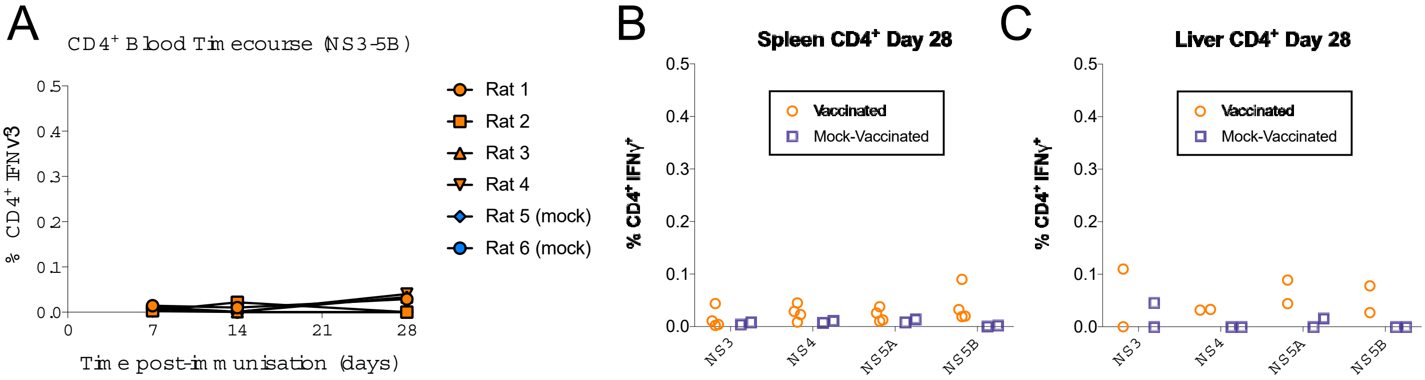

Supplemental Figure 3: CD4+ IFNγ+ cellular immune responses following vaccination of rats with an adenoviral vectored vaccine encoding the non-structural proteins of RHV (ChAd NS)

Four male Sprague-Dawley rats were vaccinated with 10^8 infectious units per dose of an adenoviral (ChAdOx1) vectored vaccine encoding the non-structural proteins of RHV. PBMCs were isolated from blood drawn weekly from vaccinated rats and stimulated with four pools of peptides representing the non-structural regions of RHV. (A) shows the time-course of CD4+ IFNγ+ responses for four weeks post-vaccination, as measured using flow cytometry, plotted are pooled responses against all four peptide pools for each rat. Mock-vaccinated rats are shown with hatched lines. (B) and (C) show the immune responses by flow cytometry four weeks post-vaccination against the four individual peptide pools as shown, for splenocytes and liver-infiltrating lymphocytes respectively, with mock-vaccinated rats shown as squares.

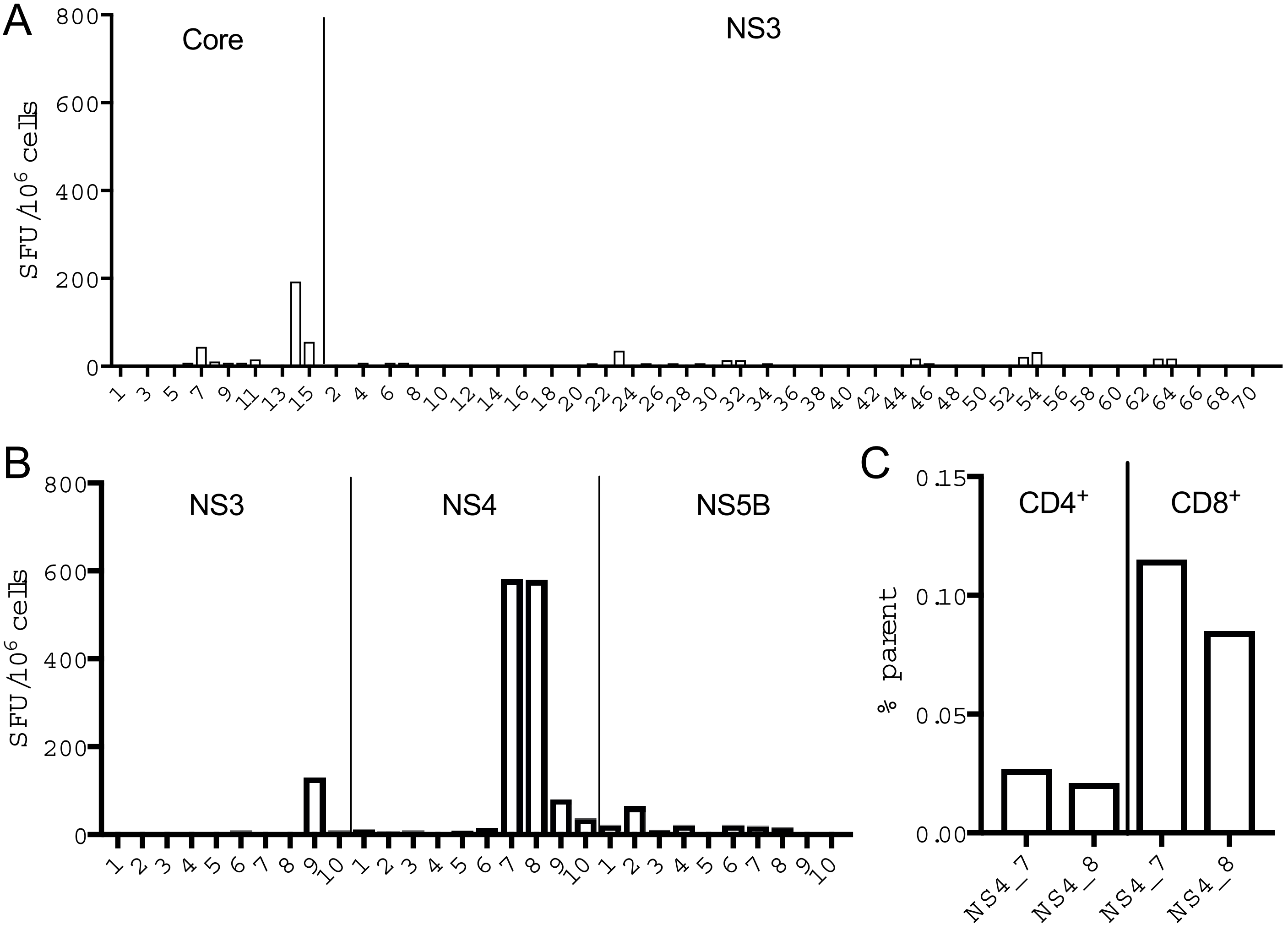

Supplemental Figure 4: Identi}401cation of immunodominant epitopes in infected and vaccinated rats

IFNv3 ELISpots and then }402ow cytometry were used on splenocytes from infected (A) and vaccinated (B) rats to identify peptides responsible for CD8$^+$ IFNv3$^+$ responses. Sub-pools of peptides and then individual peptides were screened and responses plotted as spot-forming units (SFU) per million splenocytes (ELISpot) or as % parent (}402ow cytometry, 4C) as shown.

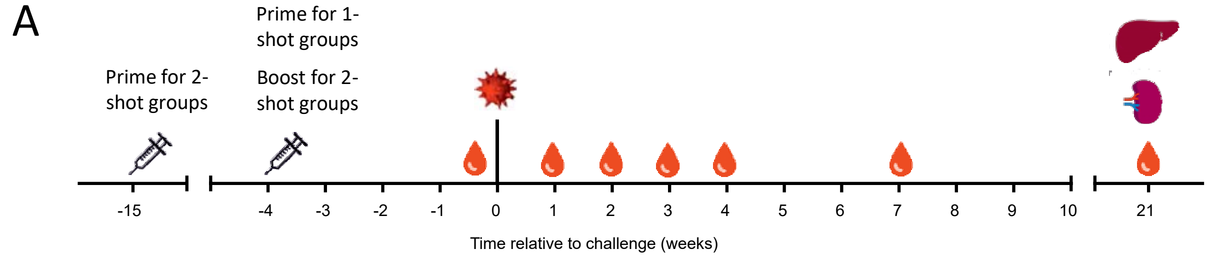

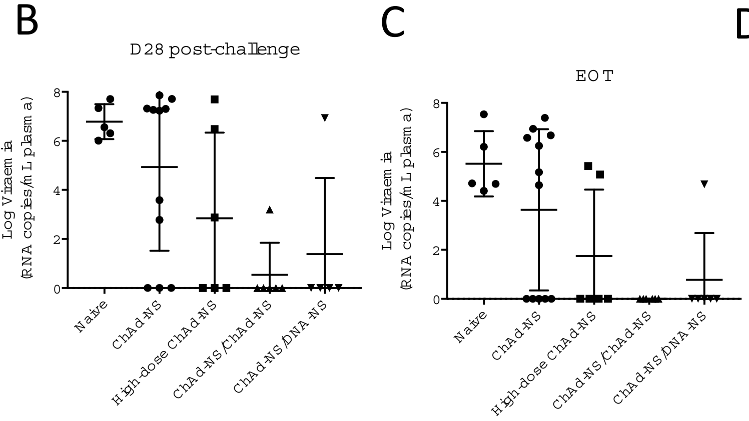

D

	ChAd-NS	ChAd-NS/ ChAd-NS	ChAd-NS/ DNA-NS	High-dose ChAd-NS
no. rats	12	6	6	6
no. cleared	5	6	5	4
% clearance	42%	100%	83%	67%
Fisher€31exact test vs naïve	0.09	0.001	0.008	0.03
no. aviraemic	0	3	2	1
% aviraemic	0%	50%	33%	17%

Supplemental Figure 5: Schema and summary of protecŸve e}403cy of ChAd-NS vaccinaŸon strategies against challenge with RHV

Male Sprague-Dawley rats (n=6 per group) were vaccinated and challenged as described in Table 1. VaccinaŸons were administered by intramuscular injecŸon into the right leg. Four weeks following vaccinaŸon RHV was administered by intravenous injecŸon into a tail vein as shown in (A). Viraemia at each Ÿme-point obtained by quanŸtaŸve PCR against a standard curve of RHV genomes of known concentraŸon. Viraemia at (B) 28 days post-challenge and (C) at end of trial (approx. 150 days post-infecŸon). (D) Summary of protecŸve e}403cy and aviraemia seen in each vaccinaŸon group.

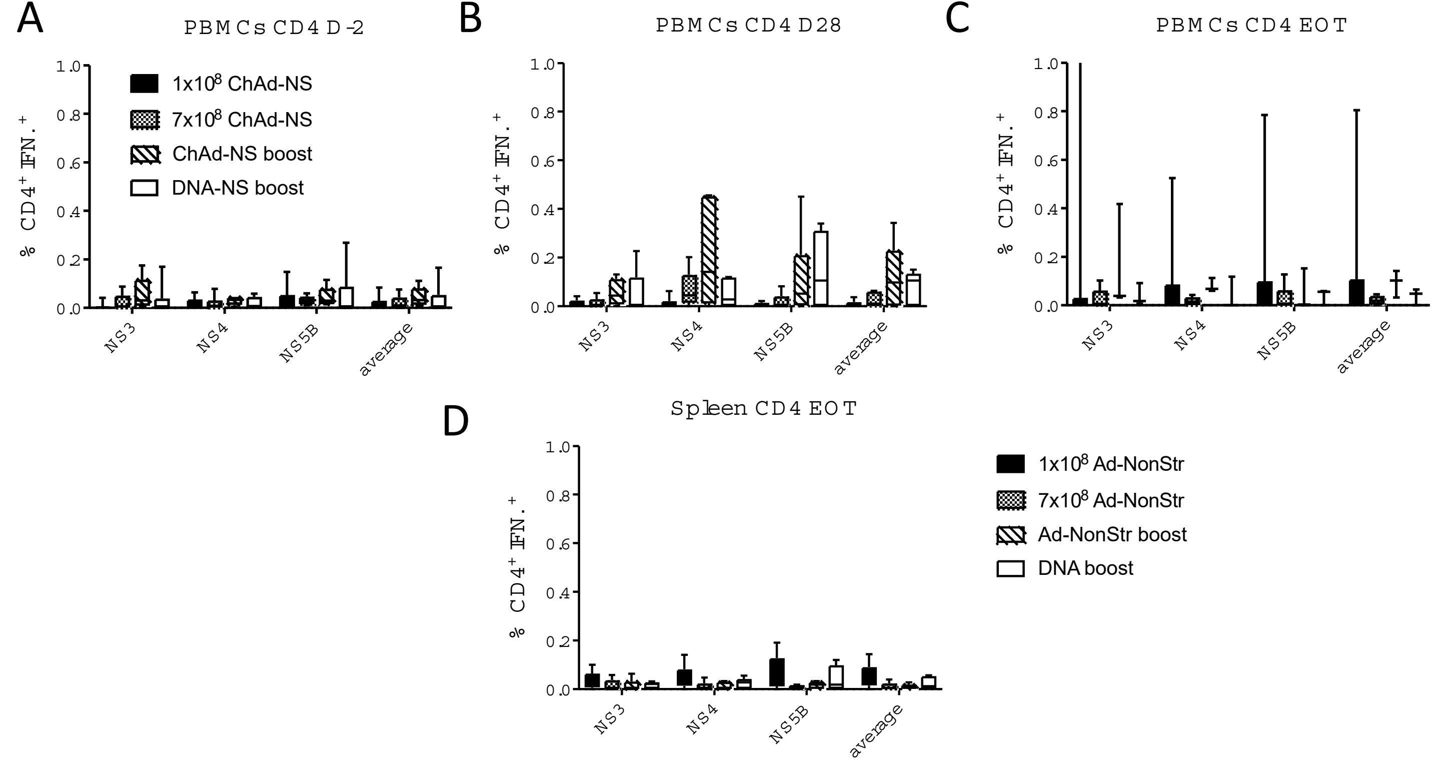

Supplemental Figure 6: E}400ects on CD4+IFN?+ cellular immunogenicity of di}400erent vaccinaŸon regimes

Male Sprague-Dawley rats (n=6 per group) were vaccinated and challenged four weeks aLer }401nal vaccinaŸon as described in Table 1. CD4+IFN?+ responses from PBMCs are shown (A) two days prior to infecŸon (B) four weeks post-infecŸon and (C) at end of study, and (D) for splenocytes at end of study.

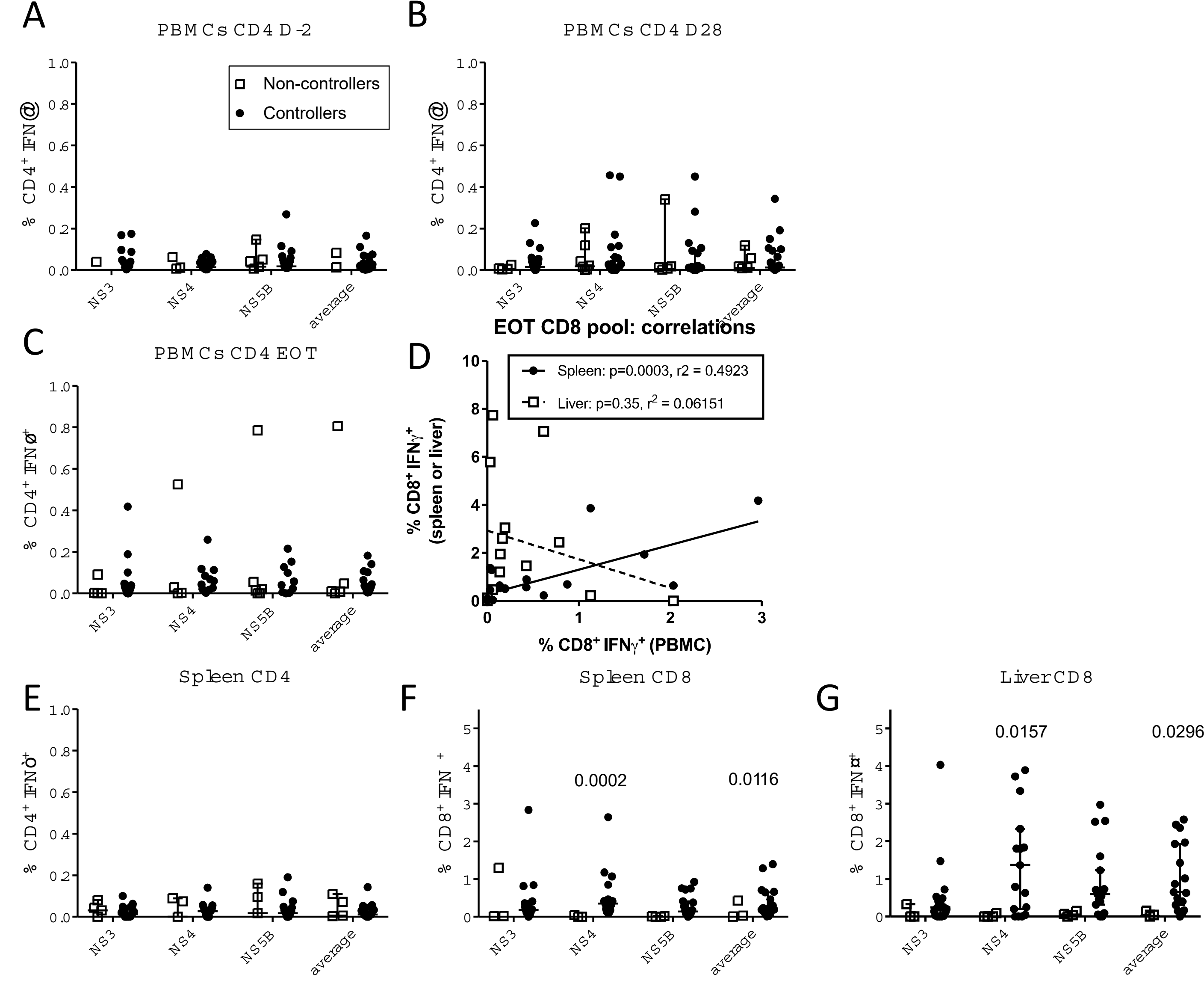

Supplemental Figure 7: AssociaŸon of CD4$^+$IFN?$^+$ and CD8$^+$IFN?$^+$ ccellular immune responses with protecŸon

Male Sprague-Dawley rats (n=6 per group) were vaccinated and challenged four weeks aLer }401nal vaccinaŸon as described in Table 1. Rats were divided into two groups, "controllers" and "non-controllers", on the basis of possessing detectable viraemia or otherwise at end of trial. AssociaŸons of CD4$^+$IFN?$^+$ responses from PBMCs are shown (A) two days prior to infecŸon (B) four weeks post-infecŸon and (C) at end of study. (D) CorrelaŸons between CD8$^+$IFN?$^+$ liver-in}401ltraŸng lymphocyte or splenocyte responses and PBMC responses at EOT. (E) CD4$^+$IFN?$^+$ and (F) CD8$^+$IFN?$^+$ responses at end of study are shown for splenocytes and (G) liver-in}401ltraŸng lymphocytes.

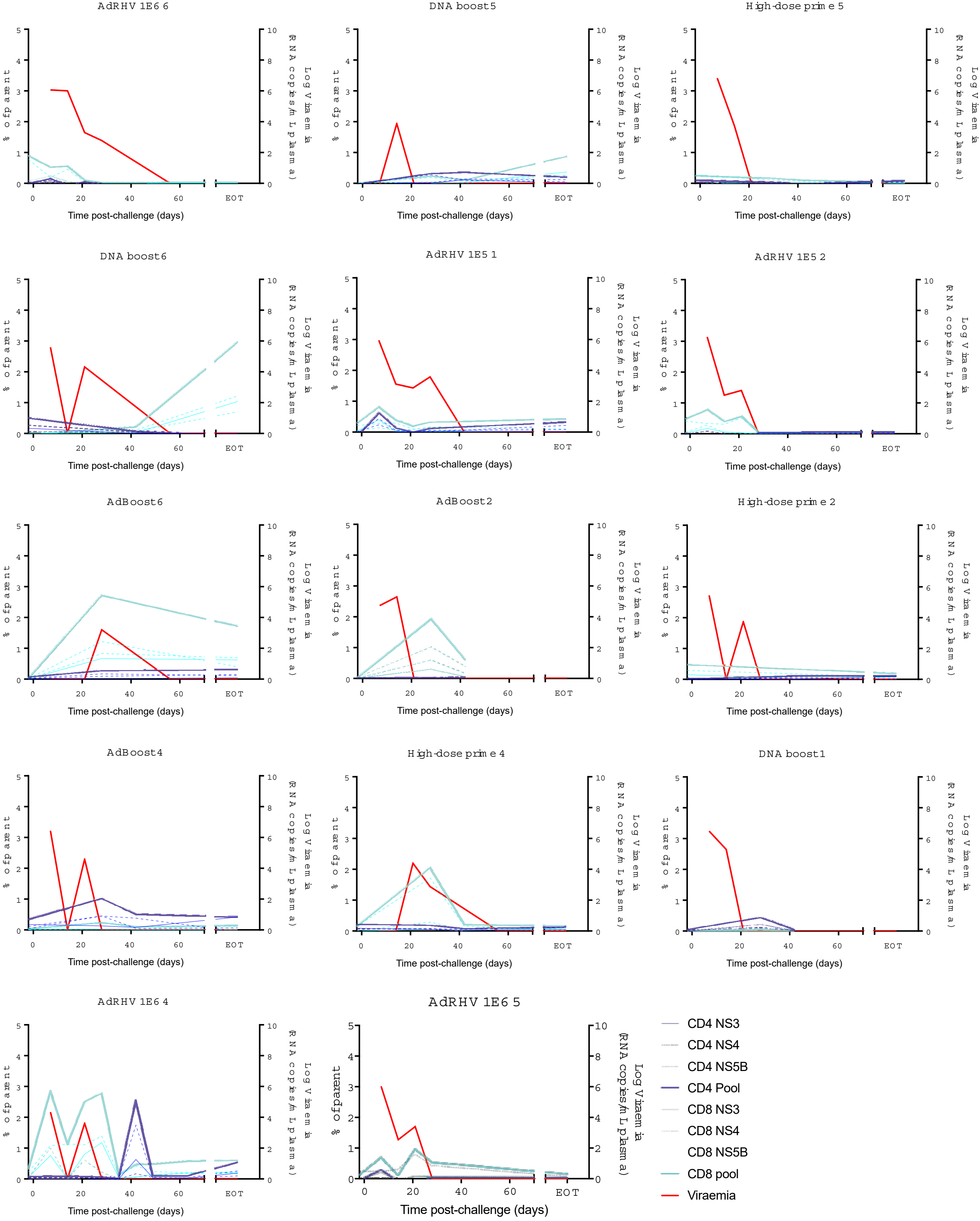

Supplemental Fig 8: Individual Ÿme-course of viraemia and blood cellular immune responses in ChAd-NS-vaccinated and challenged rats which controlled infecŸon

Male Sprague-Dawley rats were vaccinated and challenged as described in Table 2. Blood cellular immune responses were obtained at each Ÿme-point by }40w2ocytometry by sŸmulaŸon with pools of pepŸdes represenŸng three regions, NS3, NS4 and NS5B. Viraemia was obtained by qPCR against a standard curve of RHV genome copies of known concentraŸon on serum samples at indicated Ÿme-points.

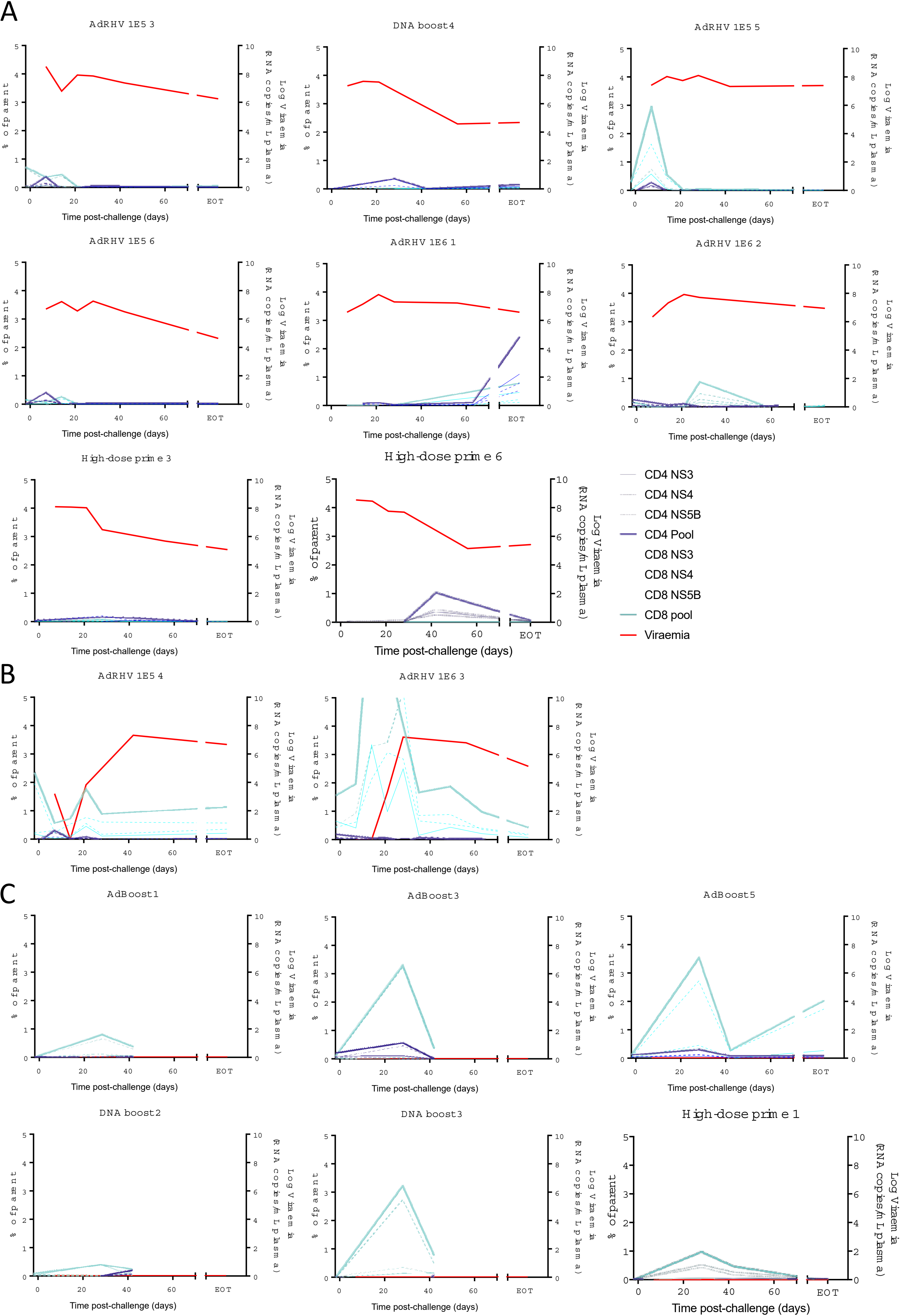

A
AdRHV 1E5 3
DNA boost4
AdRHV 1E5 5
AdRHV 1E5 6
AdRHV 1E6 1
AdRHV 1E6 2
High-dose prime 3
High-dose prime 6
% of parent
Log Viraemia (RNA copies/mL plasma)
Time post-challenge (days)
EOT
CD4 NS3
CD4 NS4
CD4 NS5B
CD4 Pool
CD8 NS3
CD8 NS4
CD8 NS5B
CD8 pool
Viraemia
B
AdRHV 1E5 4
AdRHV 1E6 3
C
AdBoost1
AdBoost3
AdBoost5
DNA boost2
DNA boost3
High-dose prime 1

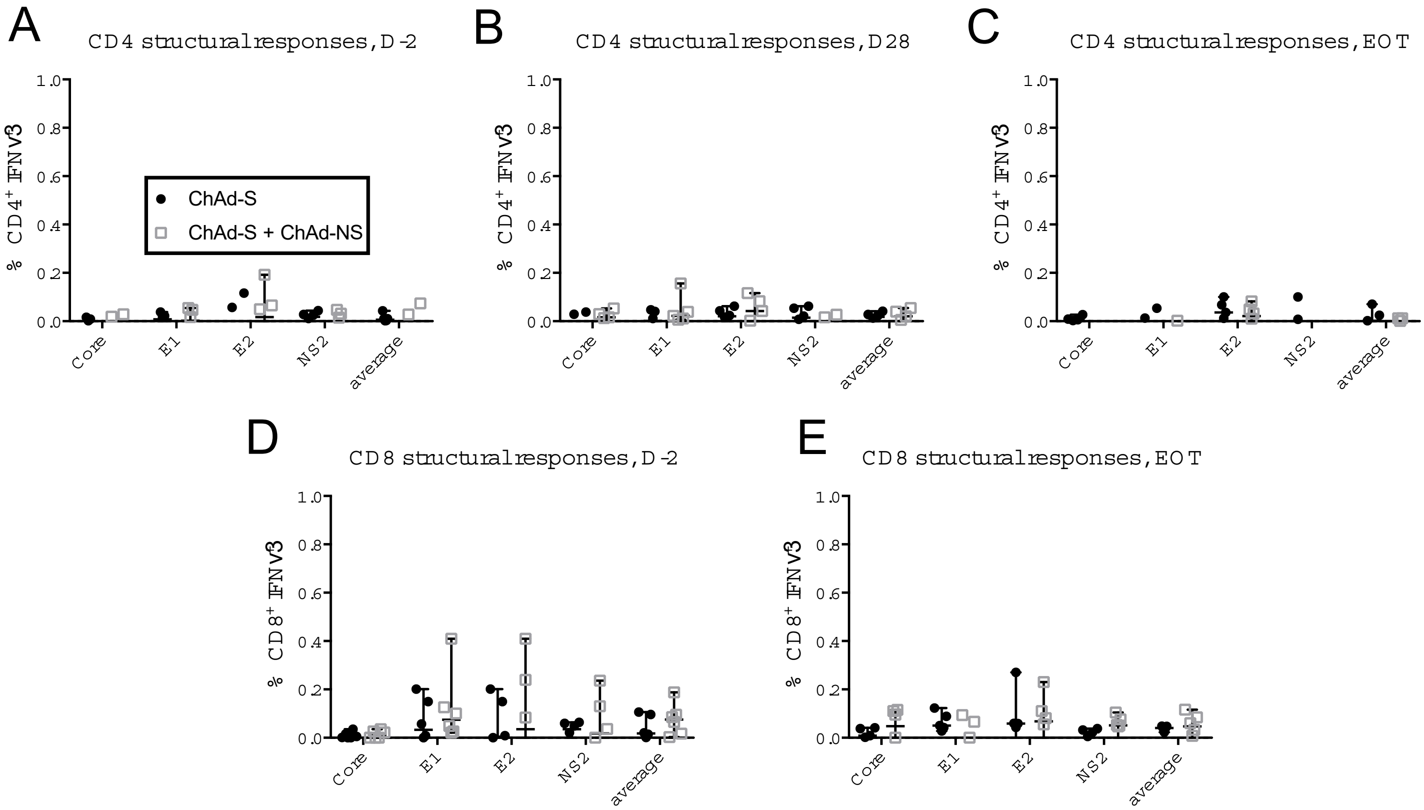

Supplemental Figure 10: Blood cellular immunogenicity against structural antigens ChAd-S alone and in combination with ChAd-NS

Male Sprague-Dawley rats (n=6 per group) were vaccinated by intramuscular injection with either ChAd-S, ChAd-NS or pre-mixed ChAd-S and ChAd-NS at a dose of 10^8 i.u. per distinct vaccine. Four weeks following vaccination rats were challenged by intravenous injection into the tail vein of 10^5 viral particles RHV. Blood cellular immune responses were compared by flow cytometry against structural antigens for single-vaccine versus combination groups for (A-C) $CD4^+IFN\gamma^+$ and (D-E) $CD8^+IFN\gamma^+$ cell types at timepoints indicated.

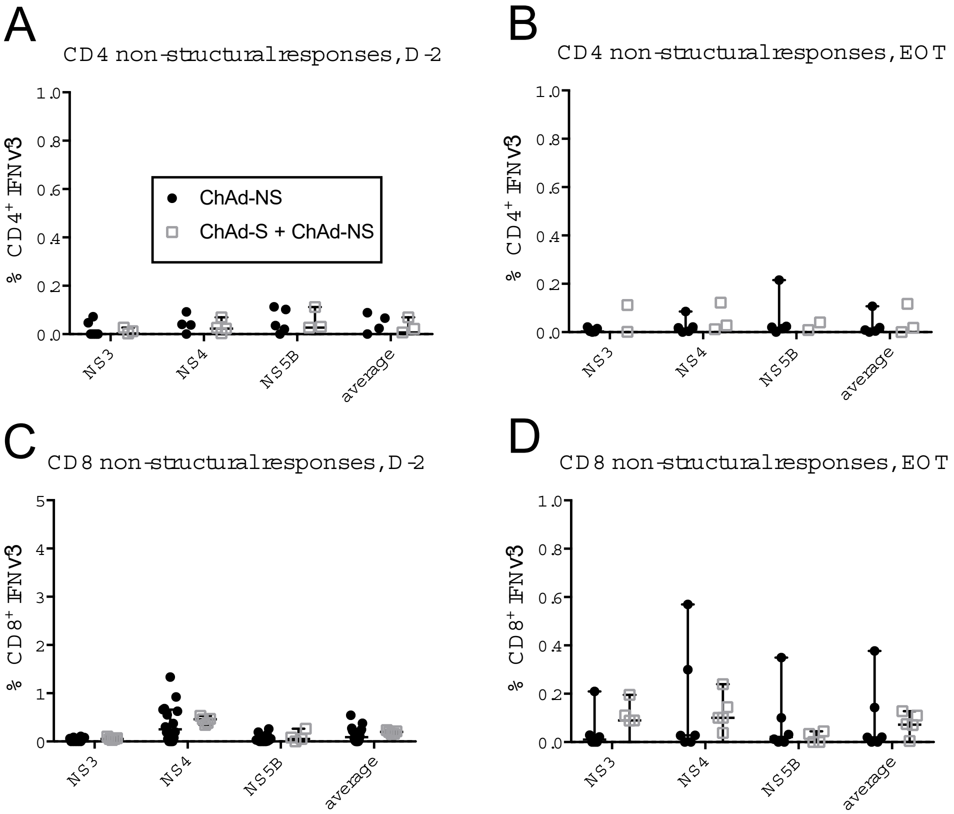

Supplemental Figure 11: Blood cellular immunogenicity against non-structural antigens of ChAd-S alone and in combination with ChAd-NS

Male Sprague-Dawley rats (n=6 per group) were vaccinated by intramuscular injection with either ChAd-S, ChAd-NS or pre-mixed ChAd-S and ChAd-NS at a dose of 10^8 i.u. per distinct vaccine. Four weeks following vaccination rats were challenged by intravenous injection into the tail vein of 10^5 viral particles RHV. Blood cellular immune responses were compared by }402 cytometry against non-structural antigens for single-vaccine versus combination groups for (A-B) $CD4^+IFN\gamma^+$ and (C-D) $CD8^+IFN\gamma^+$ cell types at timepoints indicated. Numbers above comparison groups represent p-values from ANOVA with Bonferroni's multiple comparisons test.

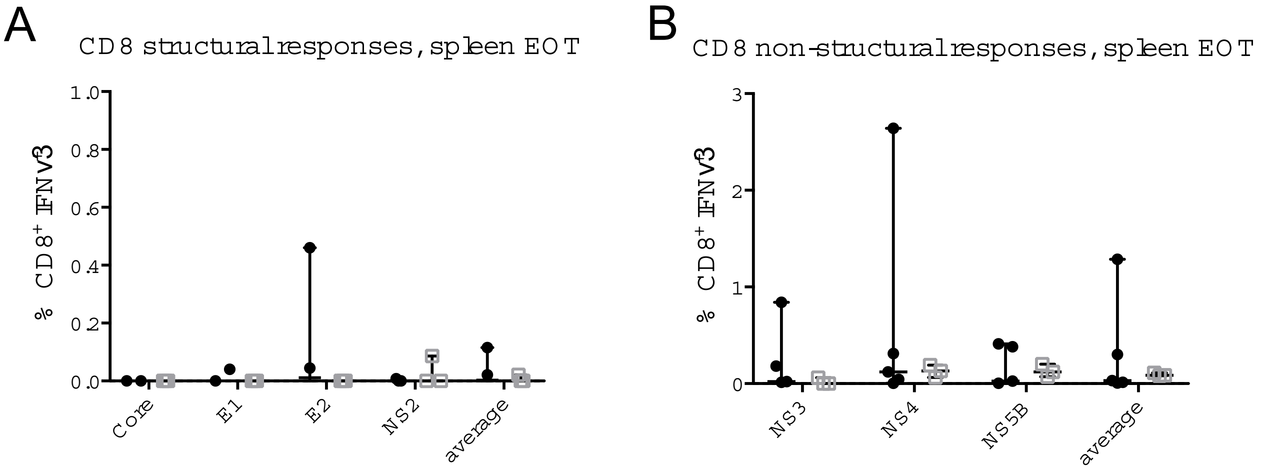

Supplemental Figure 12: Spleen cellular immunogenicity ChAd-S alone and in combination with ChAd-NS

Male Sprague-Dawley rats (n=6 per group) were vaccinated by intramuscular injection with either ChAd-S, ChAd-NS or pre-mixed ChAd-S and ChAd-NS at a dose of 10^8 i.u. per distinct vaccine. Four weeks following vaccination rats were challenged by intravenous injection into the tail vein of 10^5 viral particles RHV. CD8+ IFNv3+ Splenocyte cellular immune responses were compared by }402 cytometry against (A) structural and (B) non-structural antigens for single-vaccine versus combination groups at EOT by }402 cytometry.

Supplemental Tables

Supplemental Table 1: Sequences of immunodominant $CD8^+$ peptides from ELISpot screen

RHV protein	Peptide no.	Sequence
Core	14	GTLGWTADLLHHVPL
NS3	9	TPAEVATHLSFYHNQ
NS4B	7 & 8	AANLGAMVGHAFLTY

Supplemental Table 2: Mutations in putative T-cell epitopes in two naive and one vaccinated rat demonstrating breakthrough infection

Naïve[1]	Naïve	Vaccinated	WT[2]	Mutant	aa position
x			K K N Q T K S V L	K K N Q K K S V L	109
x		x	S A F G T V A R F	S A F G T V A R L	291
		x	S H Y I T L A A I	S H Y I T L A A V	425
x	x	x	A A V A A P V S M	A A V A A P V T K	454
x	x	x	V A N G V N T S R	V A K G V N T S R	496
		x	A S Q W A R L P G	A L Q W A R L P G	630
		x	R A V I Y I V C L	R A A I Y I V C L	745
x	x	x	P V Y A R L G K T	P V Y A R L G K S	959
		x	E V A T H L S F Y	E A A T H L S F Y	1482*
		x	M V G H A F L T Y	M A G H V L L T Y	1780*
x	x	x	P A F L W D E V E	P V F L W D E V E	2249
x	x	x	P V R Q P K P K P	P V R Q P K S K P	2351
		x	N N Y K I P V A K	N N Y K I P V A R	2861
		x	L R Y Y K R A A R	L R H Y K R A A R	2902

[1] 'x' marks mutations present in RHV isolated from that rat

[2] Residues in red denote mutation sites

Supplemental Table 3: Antibodies used in flow cytometry

	Marker/Fluorochrome	Dilution	Source
**Surface staining cocktail**	CD3-APC	1:125	ebioscience
	CD8-PerCP eFluor710	1:200	Invitrogen
	Live/Dead, Fixable Aqua dead cell stain	1:150	Life Technologies
**Intracellular staining cocktail**	CD4-APC Cy7	1:25	BD Pharmingen
	IFNγ-FITC	1:10	Biolegend
